# Chiral Plasmonic Pinwheels Exhibit Orientation-Independent Linear Differential Scattering under Asymmetric Illumination

**DOI:** 10.1021/cbmi.2c00005

**Published:** 2023-03-06

**Authors:** Lauren A. McCarthy, Ojasvi Verma, Gopal Narmada Naidu, Luca Bursi, Alessandro Alabastri, Peter Nordlander, Stephan Link

**Affiliations:** † Department of Chemistry, Rice University, 6100 Main Street, Houston, Texas 77005, United States; ‡ Department of Physics and Astronomy, Rice University, 6100 Main Street, Houston, Texas 77005, United States; § Department of Electrical and Computer Engineering, Rice University, 6100 Main Street, Houston, Texas 77005, United States

**Keywords:** linear dichroism, phase retardation, single-particle scattering spectroscopy, chiral nanoantennas, extrinsic chirality

## Abstract

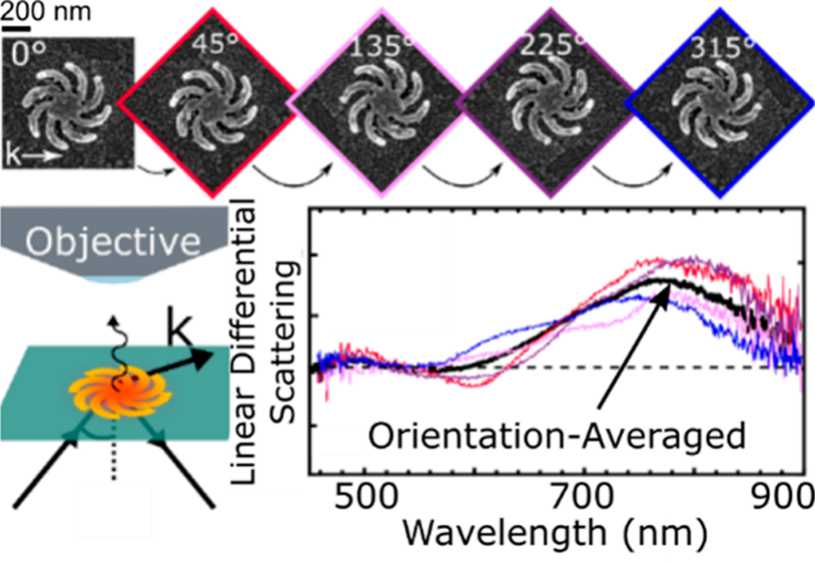

Plasmonic nanoantennas have considerably stronger polarization-dependent optical properties than their molecular counterparts, inspiring photonic platforms for enhancing molecular dichroism and providing fundamental insight into light-matter interactions. One such insight is that even achiral nanoparticles can yield strong optical activity when they are asymmetrically illuminated from a single oblique angle instead of evenly illuminated. This effect, called extrinsic chirality, results from the overall chirality of the experimental geometry and strongly depends on the orientation of the incident light. Although extrinsic chirality has been well-characterized, an analogous effect involving linear polarization sensitivity has not yet been discussed. In this study, we investigate the differential scattering of rotationally symmetric chiral plasmonic pinwheels when asymmetrically irradiated with linearly polarized light. Despite their high rotational symmetry, we observe substantial linear differential scattering that is maintained over all pinwheel orientations. We demonstrate that this orientation-independent linear differential scattering arises from the broken mirror and rotational symmetries of our overall experimental geometry. Our results underscore the necessity of considering both the rotational symmetry of the nanoantenna and the experimental setup, including illumination direction and angle, when performing plasmon-enhanced chiroptical characterizations. Our results demonstrate spectroscopic signatures of an effect analogous to extrinsic chirality for linear polarizations.

## Introduction

Chirality is a geometric property possessed by almost all biomolecules and describes the lack of inversion or mirror symmetry in a structure. Traditionally, chirality is characterized through far-field spectroscopies, but recently, chiral plasmonic substrates have gained interest for enhanced chirality sensing.^1−7^ Chiral plasmonic substrates amplify molecular chirality through a variety of mechanisms, including Coulombic interactions,^8^ providing enhanced local optical chirality,^9−13^ and employing molecular analytes to template plasmonic nanoparticles into chiral arrangements.^14,15^ Aside from the interest in chirality sensing platforms, chiral plasmonics has also emerged as a tool for achieving superior optical activity^16,17^ and negative refraction^18−24^ and is expected to lead to on-chip nanoantennas for telecommunications applications.^25−28^ As plasmonic chirality is many orders of magnitude larger than molecular chirality, it is now fairly routine to probe the chirality of single nanoparticles through circular differential scattering (CDS).^6,29−33^ Single-particle CDS affords high sensitivity for resolving enantiomeric purity^34^ and paves the way to the ultrasensitive detection of molecular chirality.^35^

Recent studies have demonstrated that even geometrically achiral plasmonic structures can exhibit an extremely strong optical activity through extrinsic chirality.^36−40^ Extrinsic chirality originates from the asymmetry of the overall experimental geometry when the mutual arrangement between the analyte and the direction of light propagation forms a chiral system.^41−44^ Extrinsic chirality has previously been reported in liquid crystals,^45^ individual carbon nanotubes,^46^ metal split ring metamaterials,^47^ single gold nanorod dimers,^48^ and fish-scale patterned arrays of wires.^49^ A key advantage of extrinsic chirality is its tunability relative to intrinsic chirality. With an achiral structure, the differential absorption and scattering under opposite circular polarizations are tuned simply by changing the angle of incidence or the relative orientation of the nanostructure and the incident light direction.^42,48,50^ Despite the well-established understanding of extrinsic chirality, the possibility of inducing linear polarization sensitivity in a rotationally symmetric structure by employing asymmetric illumination remains to be explored.

In this study, we report the observation of an extrinsic chirality analogue, in which the experimental geometry confers linear polarization sensitivity to chiral, rotationally symmetric pinwheels (PWs). We find that if the PWs are asymmetrically illuminated from a single oblique angle with orthogonal linear polarizations, the PWs exhibit extrinsic linear differential scattering (LDS) that is not observed when the PWs are symmetrically illuminated at normal incidence. However, as the PWs themselves are rotationally symmetric, the sign and intensity of the extrinsic LDS are maintained as the sample is rotated to different orientations, yielding an orientation-independent LDS. We further characterize the LDS of achiral stars as well as PWs with broken rotational symmetry caused by the removal of PW arms. These structural variants confirm that orientation-independent LDS can be observed only if the PWs are geometrically chiral and possess rotational symmetry. Finally, we characterize the differential scattering of PWs under circular and evanescent trochoidal polarizations. Although circular polarizations are insensitive to defects in rotational symmetry in the PWs due to missing arms, both linear and trochoidal polarizations can detect the orientation of these structural features. Overall, we observe orientation-independent LDS, identify the geometric requirements for observing this effect, and compare the utility of extrinsic LDS for characterizing defects in rotational symmetry to that of CDS.

## Results and Discussion

**Figure 1:**
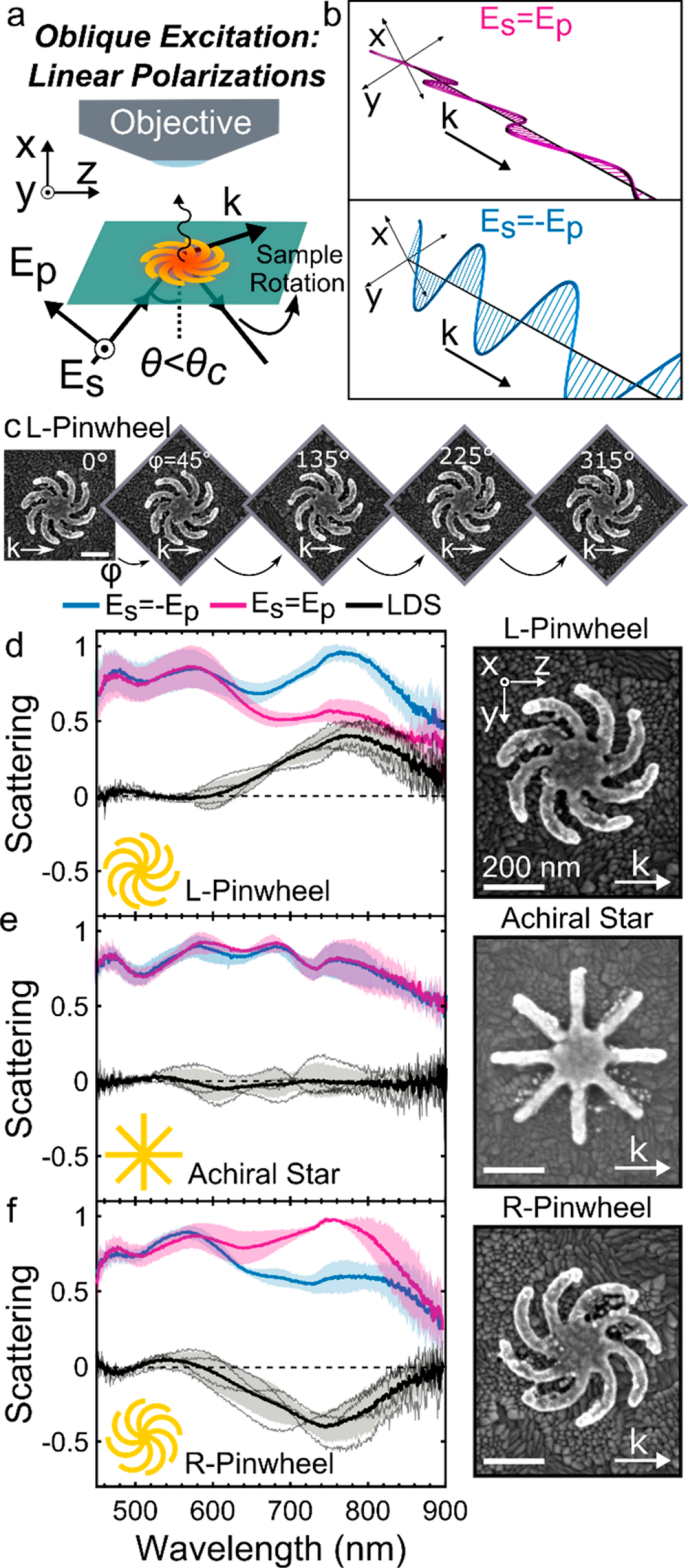
Planar chiral PWs with eight-fold rotational symmetry exhibit orientation-independent LDS under asymmetric illumination. (a) Experimental geometry for oblique incident linearly polarized excitation. *E*_s_ = ±*E*_p_ linearly polarized light is directed toward the glass–air interface at an azimuthal angle, θ, less than the critical angle, θ_c_. The refracted beam with wavevector ***k*** illuminates the PWs, and the scattered light under each polarization is collected by an objective, dispersed by a spectrograph, and then imaged using a charge-coupled device camera. The spectrograph and camera are on a mobile stage that advances across the field of view, collecting spectra in a hyperspectral fashion and capturing both the location of the PWs and their spectra simultaneously (see Methods section). (b) Instantaneous electric field distribution of oblique incident *E*_s_ = ±*E*_p_ light. (c) Scanning electron microscopy (SEM) image of an L-PW; scale bar: 200 nm. The sample is rotated through four polar angles, φ, to give the following orientations relative to ***k***: φ = 45, 135, 225, and 315°. (d) (Left) Solid colored lines: scattering from a single L-PW under oblique incident *E*_s_ = −*E*_p_ light and *E*_s_ = *E*_p_ light averaged over the four orthogonal sample orientations described in (c). The scattering spectra of the PWs are calculated by normalizing each set of polarization-resolved spectra collected at each sample orientation relative to the maximum scattering under either *E*_s_ = *E*_p_ or *E*_s_ = −*E*_p_ light, whichever is larger. Then the normalized spectra from each orientation are averaged together to give orientation-averaged spectra. See the Methods section for more details. The shaded regions represent the standard deviation of the mean scattering at each wavelength for the four orientations. Solid black line: mean LDS spectrum with shaded region displaying the standard deviation. The LDS is calculated as LDS(λ) = scatt (*E*_s_ = −*E*_p_) – scatt(*E*_s_ = *E*_p_). The light gray lines depict the LDS spectra measured at each orientation. (Right) enlarged SEM image of the L-PW showing the coordinate system and ***k***. (e,f) Same as (d) but for (e) an achiral star and (f) an R-PW.

To characterize the LDS of the PWs under asymmetric oblique illumination, we direct polarized light from a fiber-coupled broadband lamp onto a prism with an incident azimuthal angle, θ, such that the critical angle is not exceeded (Figure 1a). The transmitted light is therefore not confined to an evanescent wave and largely maintains its linear polarization. This refracted beam then irradiates the sample with wave vector ***k***. We employ PWs with eight-fold rotational symmetry so that their orientation with respect to ***k*** is poorly defined, and any pair of orthogonal linear polarizations could be used to characterize their LDS. We define LDS as LDS(λ) = scatt(γ_1_) – scatt(γ_1_ + 90°), where scatt refers to the scattering spectrum at a particular polarizer angle, γ_1_. Instead of utilizing linear polarizations along *E*_s_ (0°, perpendicular to the plane of incidence) and *E*_p_ (90°, parallel to the plane of incidence), we use *E*_s_ = ±*E*_p_ (γ_1_ = 45°, γ_1_ + 90° = 135°) linear polarizations to measure the LDS (Figure 1a,b). Our use of intermediate polarizations ensures that we do not observe LDS simply due to the differential excitation of plasmon modes within the sample plane with 0° linearly polarized excitation or perpendicular to the sample plane with 90° excitation (Figure S1).^51−54^ Finally, to assess the orientation dependence of LDS, we rotate the sample through four polar angles relative to ***k*** (45, 135, 225, and 315°) and measure the LDS at each orientation (Figure 1c).

Surprisingly, both the left-handed (L-) and right-handed (R-) PWs exhibit LDS that is independent of sample orientation and well-mirrored between enantiomers (Figure 1d,f). The L-PW preferentially scatters *E*_s_ = −*E*_p_ light from 650 to 900 nm and therefore exhibits broad, positive LDS in the red spectral region, reaching 40% of the maximum scattering at 775 nm (Figure 1d). This positive LDS is well-reproduced at all four sample orientations (thin gray lines), yielding an orientation-averaged LDS with a standard deviation of ∼10% relative to the absolute scattering at 775 nm (shaded gray region). Moreover, this orientation-independent LDS depends on the handedness of the PW, as the R-PW has negative LDS in the same red spectral region, mirroring the differential scattering of the L-PW. In contrast, the LDS magnitude of an achiral nanoantenna, a PW with straight arms or a star, is minimal at each orientation, and the observed minor LDS varies in sign as the star is rotated, resulting in negligible orientation-averaged LDS (Figure 1e). The lack of LDS for the achiral star demonstrates that, in principle, the eight-fold rotational symmetry of the PWs and the process of averaging LDS over four orientations should eliminate any linear polarization sensitivity.

The orientation-independent LDS is well-replicated over all 32 PWs characterized in this study. To quantify the magnitude of the LDS and compare across several PWs, we introduce the scattering dissymmetry, or g-factor, from the circular differential scattering literature^3,30^ and normalize the LDS relative to the average scattering under both polarizations. As we are not measuring dissymmetry or chirality here, we will simply refer to the adapted g-factor as the normalized linear differential scattering spectrum and abbreviate it as *Ln*_scat_. We calculate *Ln*_scatt_ as
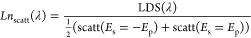
1

**Figure 2:**
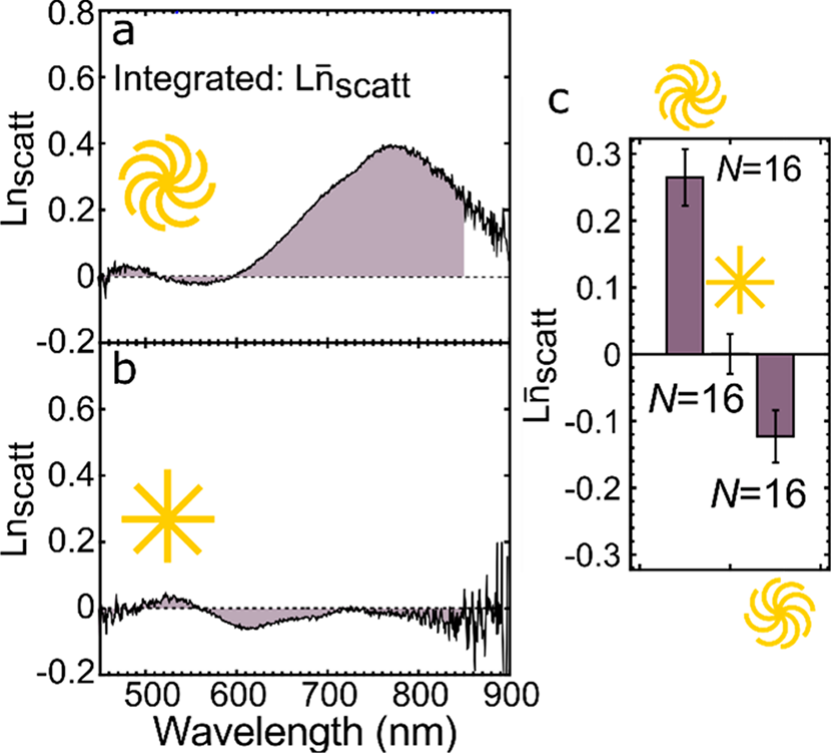
Orientation-independent LDS is reproducible over all 32 PWs characterized, whereas achiral stars yield no orientation-averaged LDS. (a) *Ln*_scatt_ spectrum calculated as described in eq 1. The shaded part denotes the region of 450–850 nm over which the integration is performed to calculate *Ln̅*_scatt_ according to eq 2. (b) Same as (a) but for an achiral star. (c) Bar chart of mean *Ln̅*_scatt_ values measured from 16 L-PWs, 16 achiral stars, and 16 R-PWs. Error bars are the standard deviations of the means.

The maximum |*Ln*_scatt_(λ)| value is 2, corresponding to scattering exclusively under one polarization at a particular wavelength. For the L-PW with LDS spectra presented in Figure 1d, we find that the maximum *Ln*_scatt_ corresponds to 0.4 at 770 nm (Figure 2a). To capture the broadband nature of the differential scattering in a single metric, we again adapt the integrated g-factor from Wilson et al.^55^ as
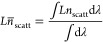
2

We integrate from 450 to 850 nm (Figure 2a, shaded region) to avoid spectral regions with significant noise (variance greater than ∼25% of the baseline). As with *Ln*_scatt_, the maximum *Ln̅*_scatt_ is 2, which would indicate that the PW scattered only under one polarization over the entire range of 450–850 nm. Employing this metric, we measure the average *Ln̅*_scatt_ for 16 L- and 16 R-PWs as 0.27 ± 0.04 and −0.12 ± 0.04, respectively (Figure 2c). In contrast, *Ln*_scatt_ for the achiral star presented in Figure 1e yields a flat spectrum (Figure 2b) with an average *Ln̅*_scatt_ of 0.00 ± 0.03 for 16 stars (Figure 2c).

Orientation-independent LDS is an unexpected observation as linear polarization sensitivities typically strongly depend on sample orientation.^56,57^ Indeed, orientation sensing of nanoparticles is a key application of LDS.^58,59^ To verify that our results are not due to errors in the experimental geometry, we confirmed that the LDS of single nanorods strongly depends on the sample orientation in our experimental setup, as expected (Figure S2). Finally, we confirmed that orientation-independent LDS is not impacted by slight polarization distortions that occur when the incident beam refracts through a prism and glass slide to irradiate the sample (Figure S3).

**Figure 3:**
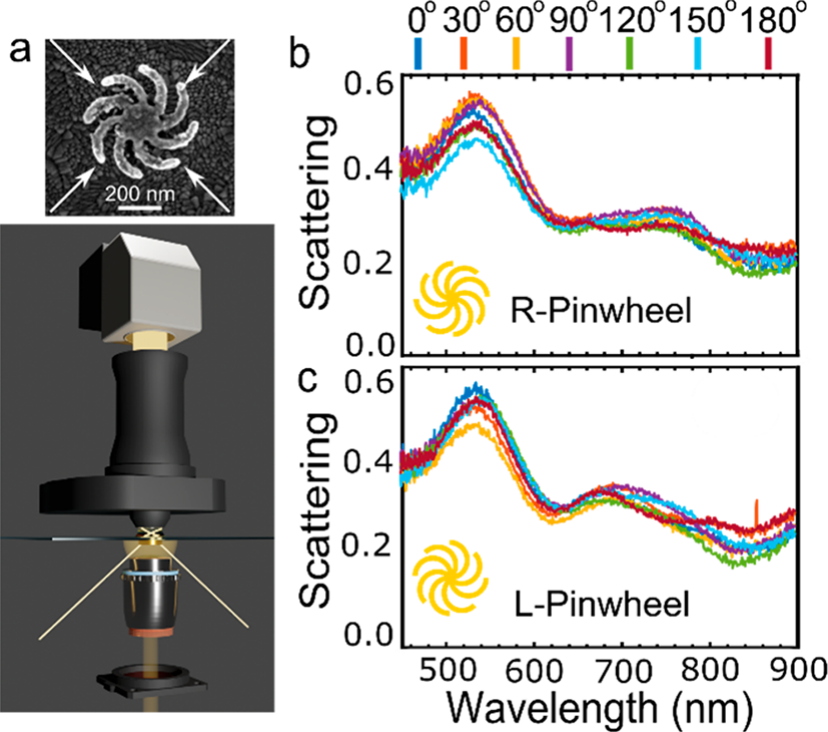
Under unpolarized, orientation-symmetric excitation from a dark-field condenser, the scattering from single PWs is not linearly polarized. (a) To characterize the possibility of the rotationally symmetric PWs exhibiting planar anisotropy, a linear polarizer is placed in the detection path of a standard dark-field scattering microscope just before the spectrograph and camera. The SEM image of an L-PW displays the four wave vectors of our oblique incidence condenser that provides orientation-symmetric illumination. (b) Scattering from a single R-PW when the detection-path linear polarizer is rotated from 0 to 180° in 30° increments. (c) Same as (b) but for an L-PW. No polarization dependence is observed, confirming that the PWs themselves do not support anisotropic scattering in the sample plane. We note that the relative amplitude of the scattering peak at 510 nm is greater under orientation-symmetric excitation than under the unidirectional excitation presented in Figure 1. The suppressed scattering intensity at longer wavelengths shown here could be due to the unpolarized rotationally symmetric excitation potentially causing interference between excited plasmons that we do not observe when exciting with polarized light from a single wave vector.

Under unpolarized, orientation-symmetric excitation from a dark-field condenser, the scattering from single PWs is not linearly polarized, underscoring the need for asymmetric illumination to observe orientation-independent LDS. Dark-field microscopes are commonly used to characterize the planar orientation of anisotropic plasmonic nanoparticles, such as nanorods, and employ illumination from an unpolarized source while positioning a rotating linear polarizer in the detection path (Figure 3a).^51,56,60^ Under unpolarized illumination, the scattering from anisotropic particles is linearly polarized, and the angle of the polarizer at which the maximum scattering occurs corresponds to the orientation of the particle (Figure S2). In contrast to linearly polarized light scattered by anisotropic nanostructures, the scattered light from the rotationally symmetric PWs does not have a significant polarization dependence under orientation-symmetric excitation. The scattering obtained from single PWs is nearly identical when the detection-path polarizer is rotated from 0 to 180° for both R- and L-PWs, as shown in Figure 3b,c. The lack of polarization dependence in the light scattered by the PWs demonstrates that, when they are evenly illuminated, they behave just as expected for rotationally symmetric, isotropic structures. Therefore, asymmetric illumination is a key requirement for observing the LDS of PWs as shown in Figure 1. Additionally, simulations of the PW scattering performed under ±45° linearly polarized excitation at normal incidence also display no LDS (Figure S4), confirming that the PWs behave isotropically, so long as the illumination does not break the rotational symmetry of the overall experiment.

**Figure 4:**
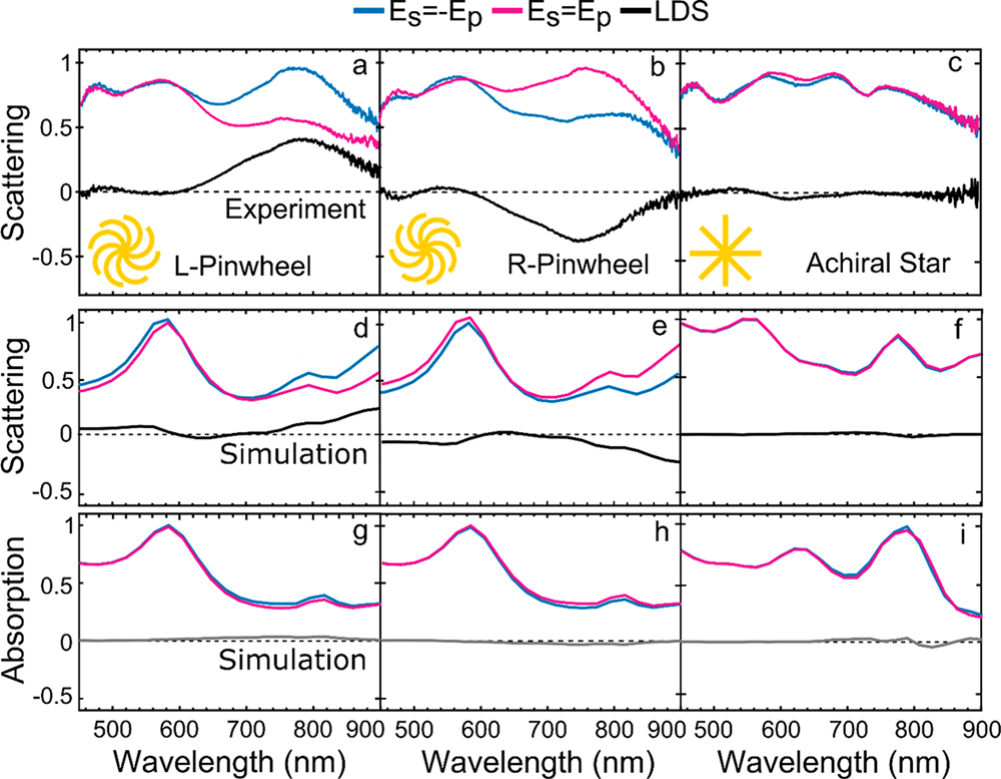
Simulated scattering spectra of the L-PW, R-PW, and achiral star under oblique incident *E*_s_ = ±*E*_p_ linear polarizations reproduce the experimental LDS while the simulated absorption shows no polarization sensitivity. (a–c) Experimental scattering spectra from a single L-PW, R-PW, and achiral star, respectively, under asymmetric illumination, averaged over the four orthogonal sample orientations described in Figure 1. (d–f) Normalized simulated scattering cross sections and LDS of the L-PW, R-PW, and achiral star with excitation conditions that mimic the experiment. (g–i) Corresponding normalized simulated absorption cross sections and the difference spectra (gray lines).

The orientation-independent LDS of the PWs is well-reproduced in COMSOL simulations performed under oblique incidence *E*_s_ = ±*E*_p_ linear polarizations. For easier comparison with the experimental data, the orientation-averaged spectra for the L- and R-PW as well as the achiral star shown in Figure 1 are replotted in Figure 4a–c. Simulated scattering of the L-PW with excitation conditions reproducing those used in the experiment confirms that the L-PW preferentially scatters *E*_s_ = −*E*_p_ light between 750 and 900 nm, yielding broad positive LDS in the red spectral region (Figure 4d). Although there are discrepancies between the experimental and simulated spectra, likely caused by complexities in the actual PW geometry due to the electron-beam lithography process compared to the idealized structures modeled, the simulated LDS generally reproduces the broad positive LDS that we measure experimentally over the same wavelength range for the L-PW (Figure 4a). Simulations of the R-PW similarly reproduce the experimentally observed negative LDS in the red-spectral region and verify the absence of LDS in achiral stars, even in the case of asymmetric illumination. Finally, simulations of the LDS of the L-PW rotated in 15° increments, instead of the 90° rotations that we perform experimentally, do not reveal a meaningful orientation dependence (Figure S5), again agreeing well with experiments.

The good agreement between measured and simulated LDS spectra allows us to gain further insights into the origin of extrinsic LDS by using electromagnetic modeling. First, simulations of the absorption reveal no evidence of a differential signal for the achiral stars (Figure 4i), as expected, but also not for the PWs (Figure 4g,h), demonstrating that the PWs do not exhibit true linear dichroism. We therefore hypothesize that the LDS in the PWs originates from interference among the excited plasmons, which selectively diminishes scattering efficiency for the PWs under particular polarizations without necessarily impacting absorption, as demonstrated next.

**Figure 5:**
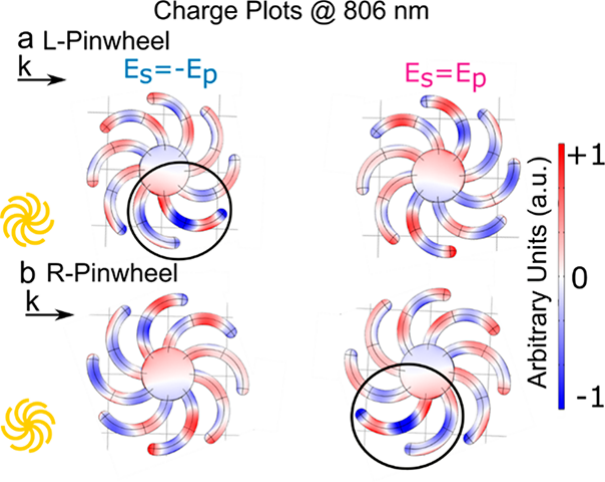
Simulated charge distributions of the PWs reveal that charges preferentially localize to particular arms, likely as a result of phase retardation. Supporting this assignment, hyperspectral LDS images reveal profiles that spatially vary in sign. (a) Simulated light-induced charge density of the L-PW at 806 nm under oblique incident *E*_s_ = ±*E*_p_ linearly polarized light excitation. (b) Same as (a) but for an R-PW.

Second, simulations of the light-induced charge density of the PWs under oblique incident linearly polarized light demonstrate that charges preferentially localize to particular arms, signifying that under asymmetric illumination the PWs do not behave isotropically as rotationally symmetric structures. Figure 5a displays the charge distributions for the L-PW under *E*_s_ = ±*E*_p_ light. Although the overall charge densities for the PW remain similar under both polarizations, supporting their nearly identical absorptions, the spatial arrangement of charges differs for each polarization. Under *E*_s_ = −*E*_p_ light, we observe increased charge density on the two PW arms circled at the bottom of the charge plot. Conversely, under *E*_s_ = *E*_p_ light, the charges do not preferentially accumulate on particular arms. Comparing these polarizations, we find that under *E*_s_ = −*E*_p_ light, the charge density of the bottom two arms is higher than that simulated for the corresponding arms under *E*_s_ = *E*_p_ light. For *E*_s_ = *E*_p_ excitation, the absence of charge accumulation and the dispersed regions of positive and negative charges are most likely a result from interfering plasmons, diminishing the net dipole moment and causing weaker scattering. For the R-PW, we note a similar differential charge pattern, with charges preferentially accumulating on the bottom two arms under *E*_s_ = *E*_p_ light, leading to a higher scattering efficiency for *E*_s_ = *E*_p_ polarization relative to *E*_s_ = −*E*_p_ light in agreement with our interpretation of the L-PW (Figure 5b). In contrast, the light-induced charge density of achiral stars does not exhibit highly localized charges under only one polarization (Figure S6). The preferential accumulation of charges on particular arms is likely enabled by phase retardation of the incoming light over the size of the PWs, allowing arms that are well-aligned with the local phase to interact favorably with the excitation.

**Figure 6:**
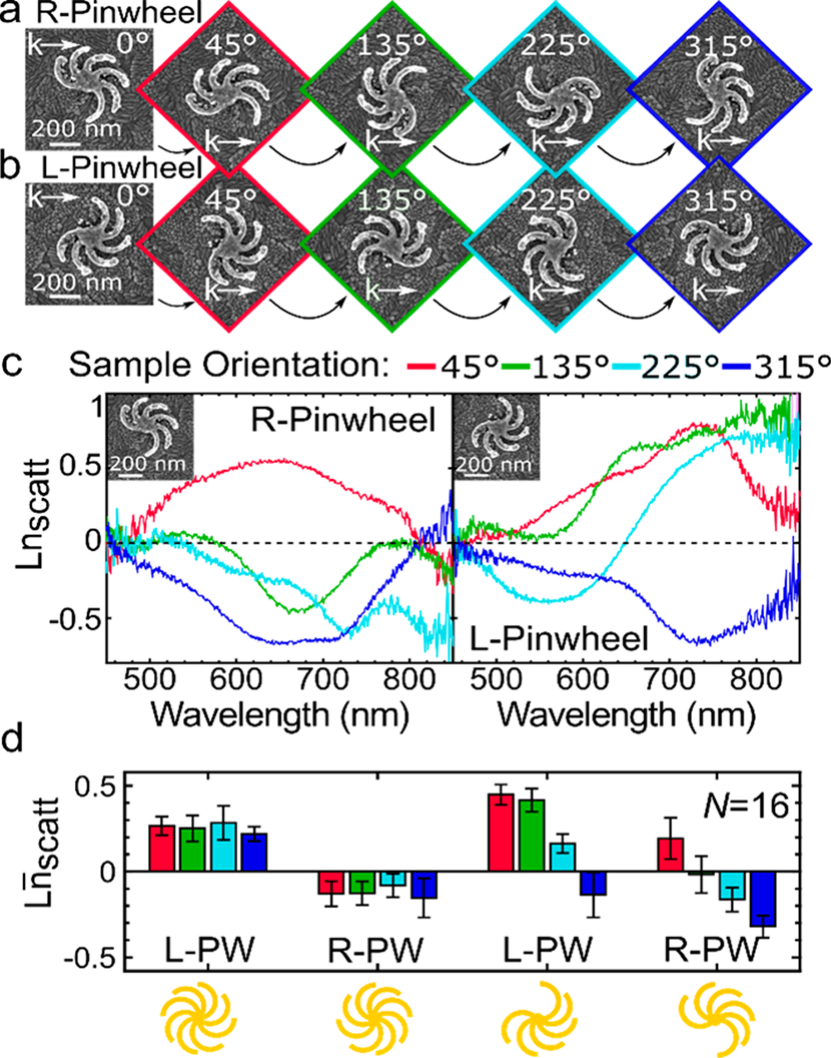
PWs fabricated with the deliberate removal of two arms exhibit inversed *Ln*_scatt_ at a single orientation. (a) SEM image of a R-PW with two arms removed. The image is rotated through each of the sample orientations to show the arrangement of the missing arms relative to ***k***. (b) Same as (a) but for the L-PW. (c) *Ln*_scatt_ spectra of the R- and L-PWs at each of the sample orientations. (d) Grouped bar chart of the mean of the *Ln̅*_scatt_ values, calculated by integrating the *Ln*_scatt_ spectra in the range of 450–850 nm (see Methods section and Figure 2) for the original 8-arm L- and R-PW and PWs with two missing arms at each of the four orientations. Error bars correspond to the standard deviation of the mean of *Ln̅*_scatt_. The cartoons below correspond to the geometry for each group in the bar chart. *N*: number of PWs considered in each bar.

After establishing the origin of extrinsic LDS for rotationally symmetric PWs, we deliberately introduce symmetry breaking by removing PW arms and find an inverse *Ln*_scatt_ at a single orientation. We employ PWs with two of the eight arms missing from the original design (Figure 6a,b) and again investigate their LDS at each of the chosen polar angles. Surprisingly, the LDS of L- and R-PWs inverts at single orientations with respect to ***k*** with the removal of just two arms. Specifically, the L-PW with two arms removed has a predominantly positive *Ln̅*_scatt_ at polar angles of 45, 135, and 225°, consistent with the LDS of 8-arm L-PWs (Figure 1) but inverts to a negative signal at 315° (Figure 6c, right). Over the 16 L-PWs with missing arms characterized, the average *Ln̅*_scatt_ at 315° is −0.14 ± 0.13, which is comparable in magnitude but opposite in sign to that of the rotationally symmetric PW (*Ln̅*_scatt_ = 0.22 ± 0.04 at 315°, Figure 6d). We attribute the increased variance in *Ln̅*_scatt_ for the 6-arm PWs relative to those of the 8-arm PWs to the orientation dependence of the LDS in the 6-arm case, potentially exaggerating effects due to subtle fabrication differences between the PWs. The negative *Ln̅*_scatt_ of the L-PW at 315° is reproduced by simulations of the differential scattering of an L-PW with two arms removed (Figure S7). Furthermore, the simulated charge distributions reveal preferential charge accumulation for the top two PW arms, which are missing at 315°, effectively reducing the scattering efficiency at this orientation (Figure S7). Similarly, the R-PW with two removed arms yields a negative *Ln̅*_scatt_ at all orientations, except for inverting to a positive LDS at a polar angle of 45° (Figure 6c, left, and 6d), again mirroring the behavior of the other chiral enantiomer. These results further confirm the importance of geometric rotational symmetry for observing orientation-independent LDS.

**Figure 7:**
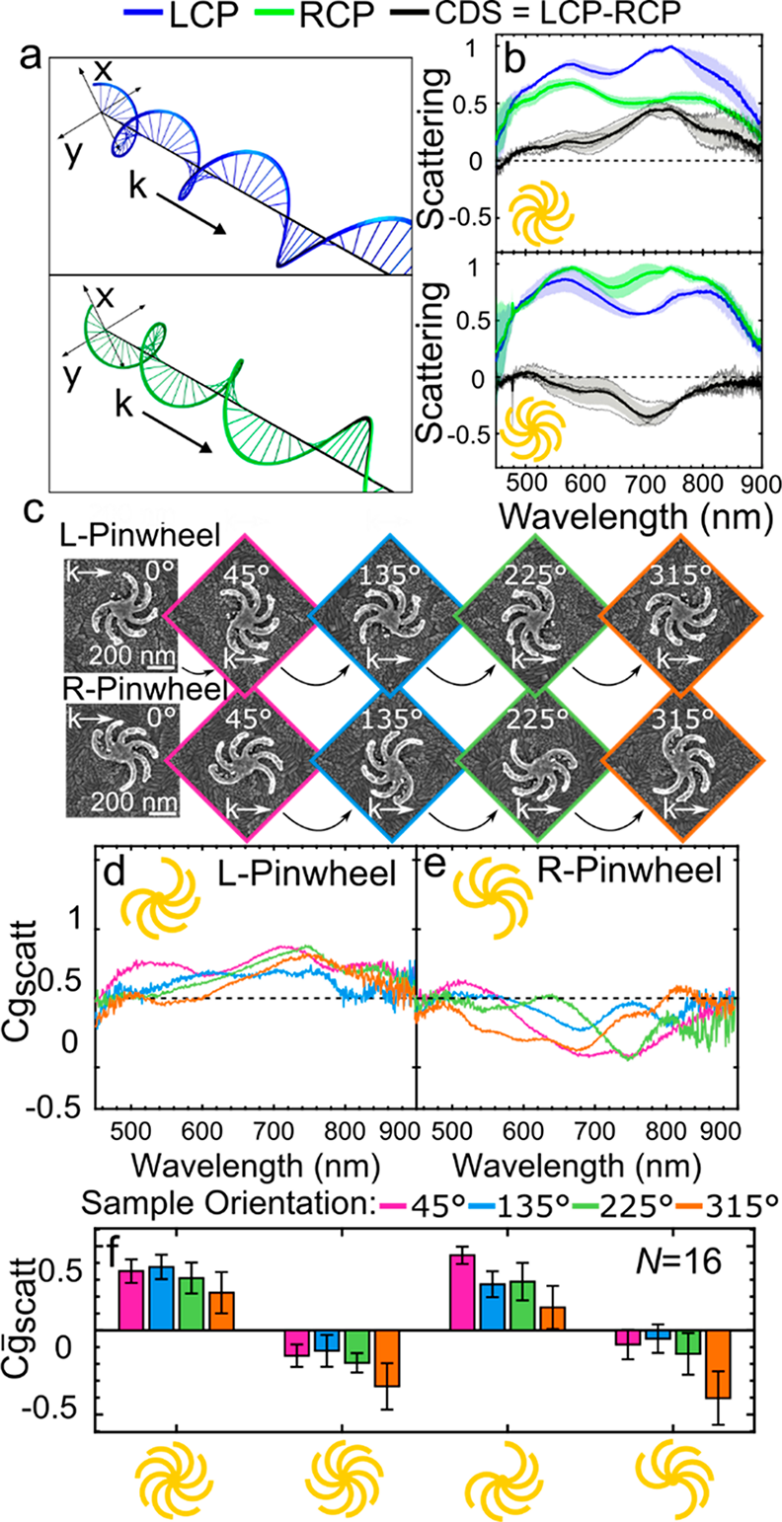
Planar chiral PWs exhibit CDS, and the sign of the CDS is maintained at each orientation, even for the PWs with two arms removed. (a) Instantaneous electric field distribution for oblique incident left- and right-handed circularly polarized (LCP and RCP) light. (b) Scattering spectra of rotationally symmetric L- and R-PWs under LCP and RCP light averaged over four orientations. The thick black line shows the averaged CDS spectrum, and the thin gray lines display the CDS spectra measured at each sample orientation. The shaded regions correspond to the standard deviation of the mean LCP, RCP, and CDS spectra for the four orthogonal sample orientations. (c) SEM images of an L- and R-PW with two arms removed rotated through each of the four sample orientations to show the arrangement of the missing arms relative to ***k***. (d,e) *Cg*_scatt_ spectra of representative L- and R-PWs with two missing arms measured at each of the sample orientations, calculated according to eq 3. The increased variance as a function of orientation observed in the *Cg*_scatt_ spectra relative to the CDS spectra in (b) may indicate that CDS is weakly sensitive to the removal of PW arms. Additionally, linear polarization components present in the oblique incident RCP and LCP excitation light likely cause an apparent orientation dependence due to the effects described in Figure 6, rather than due to true CDS. Regardless, circular polarizations do not lead to the sign reversal of *Cg*_scatt_ at a particular orientation like that observed for *Ln*_scatt_. (f) Grouped bar chart displaying the mean of the *Cg̅*_scatt_ values, obtained by integrating the *Cg*_scatt_ spectra over 450–850 nm (see Methods section) for the preserved 8-arm L- and R-PW and PWs with two missing arms, at each of the four orientations. Error bars correspond to the standard deviation of the mean values. The cartoons below correspond to the geometry for each group in the bar chart. *N*: number of PWs considered in each bar.

When characterized by circular polarizations, the sign of the CDS of the PWs is preserved at all orientations, even when two PW arms are removed. To directly compare with the LDS measurements, we employed unidirectional oblique incident circular polarizations (Figure 7a). For the preserved 8-arm case, the planar chiral PWs exhibit strong orientation-averaged CDS that is well-mirrored between enantiomers and is orientation-independent (Figure 7b, thin gray lines with shaded regions displaying the standard deviation). To compare with the *Ln*_scatt_ defined above, we calculate the scattering dissymmetry (*Cg*_scatt_) from Karst et al.^34^ as
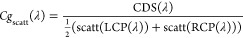
3

We then integrate *Cg*_scatt_ in the spectral range of 450–850 nm to calculate *Cg̅*_scatt_. Considering the mean *Cg̅*_scatt_ values for 16 rotationally symmetric L-PWs measured at each orientation (Figure 7f, left), the average *Cg̅*_scatt_ for the four orientation angles exceeds that of *Ln̅*_scatt_ (*Cg̅*_scatt_ = 0.32 ± 0.07; *Ln̅*_scatt_ = 0.26 ± 0.03). The observed CDS of the PWs is expected on the basis of the planar chirality of its geometry,^50^ but intriguingly, the *Cg̅*_scatt_ of the PWs is only 23% greater than the *Ln̅*_scatt_, despite the high degree of rotational symmetry of the PW. In stark contrast to the LDS case, when two PW arms are removed and the CDS is again measured at each of the chosen sample orientations, as shown in Figure 7c, the sign of the *Cg*_scatt_ spectra does not invert at any orientation for either enantiomer (Figure 7d,e). Indeed, the average *Cg̅*_scatt_ for 16 PWs with missing arms is positive at each orientation for the L-PWs and negative for the R-PWs (Figure 7f, right), consistent with the behavior of the 8-arm symmetric PWs (Figure 7f, left). Taken together, the preserved sign of the CDS from the PWs, even after symmetry breaking, confirms that the handedness of the structures does not meaningfully change as the rotational symmetry is broken. Therefore, we conclude that circular polarizations provide no true sensitivity to disruptions in rotational symmetry in the PWs, while the extrinsic LDS strongly depends on the orientation of the missing arms.

Finally, we find that evanescent trochoidal polarizations could provide even greater sensitivity for detecting defects in the rotational symmetry (Figure S8). To achieve clockwise and counterclockwise trochoidal excitation, we utilize the total internal reflection of *E*_s_ = ±*E*_p_ linearly polarized light at the glass–air interface. This excitation geometry yields a surface-confined evanescent wave with planar rotational, trochoidal field motion.^61,62^ To characterize the trochoidal polarization sensitivity of the PWs, their scattering under clockwise and counterclockwise trochoidal polarizations is collected and then subtracted to yield the trochoidal differential scattering (TDS) spectrum. We find that the TDS of PWs with missing arms also inverts at a single sample orientation but yields an even stronger signal than LDS. For example, the L-PW with two missing arms yields a *Tn̅*_scatt_ at a 315° polar angle of −0.55 ± 0.17, which is nearly three times greater than the corresponding *Ln̅*_scatt_ (−0.20 ± 0.15) measured at the same orientation (Figure S8). The increased polarization sensitivity observed under trochoidal polarizations is likely due to a combination of two factors: increased phase retardation in the evanescent wave relative to the oblique angle excitation and true trochoidal polarization sensitivity of the PWs. These results suggest that TDS may outperform extrinsic LDS as a method for characterizing defects in the rotational symmetry.

## Conclusion

In conclusion, asymmetric illumination from a single oblique angle can induce orientation-independent linear polarization sensitivity in chiral PWs with high rotational symmetry. We find that this effect is mediated by phase retardation, allowing charges to localize to a subset of PW arms and supporting plasmon oscillations that either constructively or destructively interfere depending on the handedness of the PW and the polarization. Examination of achiral stars and PWs with two arms removed, and thus lifted rotational symmetry, reveals that orientation-independent LDS strictly requires a chiral, rotationally symmetric structure. Furthermore, we note that extrinsic LDS is highly sensitive to the orientation of rotational symmetry defects in the PWs, while traditional chiroptical spectroscopies such as CDS are not, suggesting an interesting application of this technique. Overall, our results highlight the necessity of considering the overall rotational symmetry of both the nanostructure and the illumination geometry when performing chiroptical spectroscopies in order to suppress linear artifacts. Moreover, we expect that nanoantennas exhibiting an orientation-independent LDS may find use as position-invariant linear polarization switches in nanophotonic circuits. Ultimately, extrinsic LDS forms a linear analogue to extrinsic CDS, revealing additional and unexplored flexibilities in linear polarization spectroscopies.

## Methods

### Single-Particle Spectroscopy

For unidirectional oblique incident excitation measurements, scattering spectra from single PWs were collected on an inverted dark-field microscope (Ziess, Axiovert 200 MAT) using a 40× objective (Zeiss, NA = 0.6). Due to collection angle and working distance constraints, a higher NA objective could not be used. However, the 40× objective still allowed us to capture all of the relevant scattering required to characterize the LDS for the L- and R-PWs (Figure S9). The excitation path was composed of a fiber-coupled quartz tungsten halogen lamp (Newport 66884) with the fiber output mounted in a rail assembly. The rail assembly contained an achromatic 3 cm lens for collimation (Thorlabs AC254-030-AB), an infrared filter to protect the film polarization optics from overheating (Thorlabs FGS550), a linear polarizer (Thorlabs LPVIS100), and another 3 cm lens to focus the output light (Thorlabs AC254–030-AB). The focused light from the assembly was directed toward a 20 mm borosilicate glass equilateral prism (OptoSigma DPB-20-10H) at a ∼27° angle. For experiments with linearly polarized excitation, the linear polarizer was set to ±45° relative to the *E*_p_ axis (Figure 1a). For experiments with circular polarizations, a quarter-wave plate (Edmund Optics no. 63-935; effective range of 610–850 nm) was placed in the rail assembly after the linear polarizer, and the linear polarizer was set to ±45° relative to the fast axis to obtain LCP and RCP light, respectively.

In the detection path, a 20 μm slit aperture spatially filtered the scattered light at the first image plane of the microscope, allowing the signal to be collected in a hyperspectral fashion by moving a spectrograph (Acton SpectraPro 2150i) and a charge-coupled device camera (Pixis 400BR) mounted on a translation stage. The translation stage was advanced over the field of view by a linear actuator (Newport LTA-HL), while spectrally resolved slices were collected at each step, building up a data cube composed of the spatial location of each PW with complete spectral information in the third dimension.

For all prism-coupled experiments, the sample was mounted on a circular stage insert made in-house with milled markings placed every 15°. At the start of the experiment, the array was aligned to the charge-coupled device camera such that the maximum scattering intensities of PWs in a single row of the lithographic array were contained within just one or two rows of pixels. This orientation was assigned as 0°. Then the sample was rotated 45° counterclockwise, as shown in Figure 1a, and polarization-resolved hyperspectral images were acquired. The sample was then rotated 90° counterclockwise three times, and polarization-resolved hyperspectral images were again collected at each orientation. In total, hyperspectral imaging was performed with the sample orientated at 45, 135, 225, and 315° relative to the 0° alignment performed at the beginning of the experiment.

For experiments employing condenser excitation, an inverted dark-field microscope (Zeiss, Axio Observer, m1) was used. Light from a tungsten halogen lamp (Zeiss, Axioline HAL) was focused by using the condenser onto the sample at a 36° angle. The scattered light from the PWs was collected in the same manner as that described above. The incident light was unpolarized, but a linear polarizer (Thorlabs LPVIS 100) was placed in the detection path as described in the relevant figure captions.

### Analysis

Scattering spectra were analyzed by custom MATLAB R2017a scripts that performed background subtraction, white light correction, and dark count correction. The background correction was implemented by averaging spectra from 10% of pixels with the lowest intensity in each hyperspectral image. The averaged background was then subtracted from each pixel in the hyperspectral image. This background correction was performed separately for each image and for each polarization in order to account for any polarization dependence of the background. Each spectrum, unless otherwise noted, was calculated by summing the spectra from a 7 × 7 square around the particle, and 3 spectra for each particle at each polarization were averaged together. Aberrant spikes in the data from cosmic radiation were removed through the “filloutliers” MATLAB function, which applies a ∼20 nm moving window across the spectra to search for outliers, defined as three standard deviations away from the mean in each window, and uses a shape-preserving piecewise cubic interpolation to fill any outliers found. The averaged and de-spiked spectra were then normalized relative to the maximum intensity of either of the two polarizations used, whichever is the larger value. Normalization was performed at each sample orientation so that the LDS at all sample orientations can be accurately compared, as slight differences in the alignment of the excitation at each orientation could not be avoided. The integrated *Ln̅*_scatt_, *Cg̅*_scatt_, and *Tn̅*_scatt_ values were calculated by numerical integration of the *Ln*_scatt_, *Cg*_scatt_, and *Tn*_scatt_ spectra, respectively, over the spectral range of 450–850 nm using the “trapz” function in MATLAB.

### Electron-Beam Lithography

All samples were fabricated by using electron-beam lithography. The PW arms were designed with a 100 nm inner radius of curvature, a 40 nm arm width, and a 150° span angle. PWs with missing arms retained the same dimensions as above with a semicircle added to the center to retain a symmetric ∼110 nm diameter disc in the center of the PW (Figures 6 and 7). This disc diameter was estimated from the designed size of the central region where the arms meet in the 8-arm PWs. Achiral stars were composed of 40 nm × 240 nm rods and arranged in a rotationally symmetric fashion, just as the PWs. The length of 240 nm was chosen to match the average of the outer and inner diameters of a single PW arm.

Samples were prepared on indium tin oxide coated float glass (Delta Technology LTD CG-50IN-S107) cleaned in 3 sequential 10 min sonication steps of a 2% v/v solution of Hellmanex detergent and Milli-Q water, Milli-Q water, and 200 proof ethanol. The substrates were then oxygen-plasma cleaned for 2 min immediately before spin-casting poly(methyl methacrylate) (Kayaku Advanced Materials, 950 PMMA A4) at 4000 rpm for 60 s. After baking on a hot plate at 180 °C for 2 min, the substrates were taken to an Elionix ELS-G100 operating at 1 nA and 100 kV. The patterns for the PWs and stars were written at doses varying from 1200 to 1600 μC/cm^2^ and were developed in a 1:3 isopropyl alcohol/methyl isobutyl ketone solution for 65 s and rinsed in isopropyl alcohol for 30 s before being gently blown dry with nitrogen. The developed samples were then transferred to an electron-beam evaporator, where 2 nm of titanium and 40 nm of gold were evaporated onto the sample at a rate of 0.05–0.15 nm per second. Finally, the samples were left in acetone for at least 36 h and gently sonicated to complete lift-off before being gently dried with nitrogen.

### Theory

All electromagnetic calculations were performed by using the commercial software COMSOL Multiphysics. The simulated structures were modeled in close agreement with the experimental geometric parameters (Figure S10). Each structure was placed in a 1400 nm thick air layer of refractive index 1, lying on the surface of a 400 nm thick background medium layer, with both dimensions along the vertical direction. An effective value of 1.77 was used for the refractive index of the background medium, which represents an intermediate value between the refractive index of glass (1.52) and that of the 120–160 nm thick indium tin oxide layer (1.88; more details can be found in our previous work^50^). The 2 nm Ti adhesion layer was neglected in the calculations. The lateral dimensions of the simulated domain were 1400 nm. The simulation domain was truncated by exploiting 50 nm thick perfectly matched layers in all of the spatial directions. Each structure’s optical properties were described by using the Rakić permittivity for gold.^63^ The incident light was linearly polarized, reaching the target through the background medium with an incident angle of 39° to the surface normal, below the critical angle needed for total internal reflection at 42° at an air–glass interface. Absorption and scattering spectra were obtained by performing the electromagnetic simulations explicitly for 25 wavelengths in the spectral region of interest and then interpolating them using cubic spline functions. The scattering spectra were calculated by integrating the Poynting vector scattered by the structure on the surface of a virtual hemisphere, enclosing the whole structure, located within the air layer and centered in the middle of the structure’s surface that sits on the planar interface between the air and background medium layers. The absorption spectra were obtained by integrating the total power dissipation density in the structure. The charge density plots were generated by taking the potential gradient normal to the surface and multiplying it by the permittivity at the frequencies of the selected resonance peaks.

## Supporting Information Available

Polarization analysis of light scattered from PWs under unpolarized oblique incidence excitation, LDS analysis of single nanorods, LDS measurement performed without refraction of the incident beam, simulated LDS of PWs under normal incidence excitation, simulated LDS of an L-PW at different PW orientations, charge plots of an achiral star, simulated LDS and charge plots of an L-PW with two missing arms, trochoidal differential scattering analysis of PWs, simulated comparison of the PW LDS using different numerical aperture objectives, description of the simulation geometry (PDF)

## Abbreviations

CDS: circular differential scattering
LDS: linear differential scattering
PW: pinwheel
R-PW: right-handed PW
L-PW: left-handed PW
SEM: scanning electron microscope
scatt: scattering
