# Supplementary material for: Chiral Plasmonic Pinwheels Exhibit Orientation-Independent Linear Differential Scattering under Asymmetric Illumination
Source: Chem Biomed Imaging. 2023 Mar 6;1(1):30–9. doi: 10.1021/cbmi.2c00005 (PMC10131493; doi:10.1021/cbmi.2c00005)
Supplement: Supplementary file 1 — im2c00005_si_001.pdf [file im2c00005_si_001.pdf]

Supporting Information

Chiral-Plasmonic Pinwheels Exhibit Orientation-Independent Linear Differential Scattering Under Asymmetric Illumination

Lauren A. McCarthy[1,#,†], Ojasvi Verma[1,#], Gopal Narmada Naidu[2], Luca Bursi[2,‡], Alessandro Alabastri[3], Peter Nordlander[2,3], Stephan Link [1,3]*

[1]Department of Chemistry, Rice University, 6100 Main St., Houston, TX, 77005, USA

[2]Department of Physics and Astronomy, Rice University, 6100 Main St., Houston, TX, 77005, USA

[3]Department of Electrical and Computer Engineering, Rice University, 6100 Main St., Houston, TX, 77005, USA

[#] L. A. McCarthy and O. Verma contributed equally to this work.

[†]L. A. McCarthy present address: Department of Chemistry, University of Michigan, 930 N. University Ave., Ann Arbor, MI 48109

[‡]Present address: Dipartimento di Fisica, Informatica e Matematica-FIM, Università di Modena e Reggio Emilia, and Istituto Nanoscienze, Consiglio Nazionale delle Ricerche CNR-NANO-S3, I-41125 Modena, Italy

* E-mail: slink@rice.edu

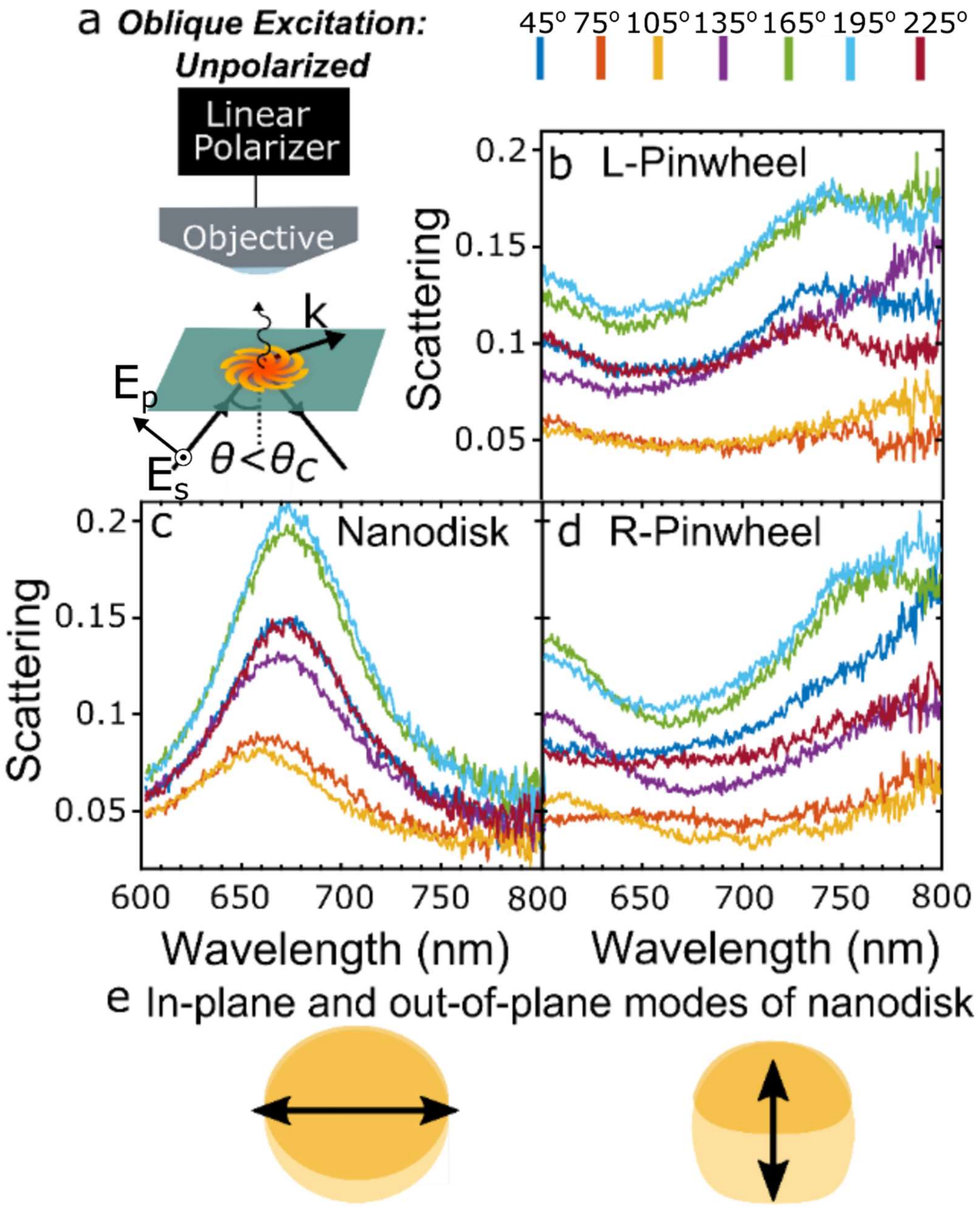

Figure S1: Light scattered from L-pinwheels (PWs), R-PWs, and disks all have a similar polarization dependence when irradiated with unpolarized light from an oblique angle due to the differential excitation of out-of-plane and in-plane plasmon modes. (a) Experimental geometry with unpolarized excitation and a linear polarizer in the detection path. E_s and E_p, as in the Main Text, are the incident light polarization components parallel and perpendicular to the

plane of incidence. Scattering spectra from single particles as the detection-path polarizer is rotated from 45° to 225° for (b) an L-PW, (c) a nanodisk, and (d) an R-PW. The spectra all show a similar polarization dependence, strongly scattering when the polarizer angle is near 180° (165°, green line, and 195°, teal line) and weakly scattering when the polarizer is near 90° (75°, orange line, and 105°, yellow line). This polarization dependence from an unpolarized source is likely due to the asymmetric illumination differentially exciting the in-plane and out-of-plane plasmon modes shown conceptually in (e). Specifically, although the incident light is unpolarized and contains both E_s and E_p components in equal amounts, its E_s components strongly couple with the in-plane modes of the nanostructure oriented at ~180° as the electric field of E_s polarized light lies only in the sample plane.[1] Meanwhile, the E_p components of the illumination can couple with both in-plane and out-of-plane modes as the electric field of E_p polarized light lies partially out of plane. The polarization of the out-of-plane mode does not register with the linear polarizer in the detection path as this method only characterizes the two-dimensional projection of the polarization state of the scattered light and cannot measure out-of-plane effects.[2] The in-plane component excited by E_p polarizations is oriented perpendicular to that excited with E_s, at 90°, but is reduced in magnitude as the intensity of this polarization component is split between the in- and out-of-plane modes.

To avoid preferentially coupling to in-plane or out-of-plane components when exciting with linearly polarized light in our linear differential scattering (LDS) measurements, we utilize the intermediate polarizations of $E_s = \pm E_p$, corresponding to 45° and 135°. Indeed under unpolarized excitation, with the detection-path polarizer set to 45°, 135°, and 225° (blue, red, and purple lines), the scattering intensity of each nanostructure is nearly identical, confirming no preferential coupling with in-plane and out-of-plane modes at intermediate polarizations.

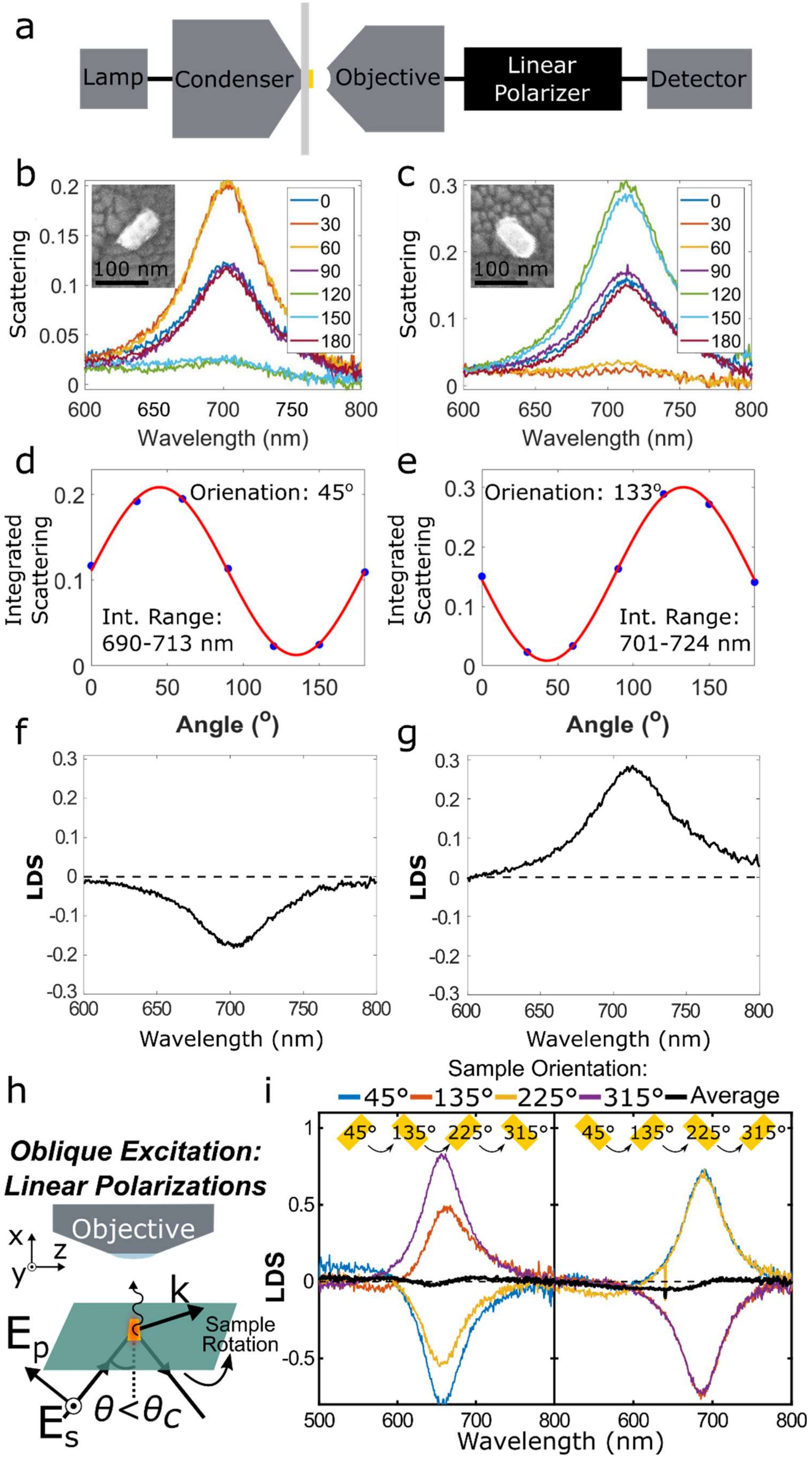
a
Lamp
Condenser
Objective
Linear Polarizer
Detector
b
c
Scattering
100 nm
0
30
60
90
120
150
180
Wavelength (nm)
d
e
Integrated Scattering
Orienation: 45°
Orienation: 133°
Int. Range: 690-713 nm
Int. Range: 701-724 nm
Angle (°)
f
g
LDS
Wavelength (nm)
h
Oblique Excitation: Linear Polarizations
Objective
x
y
z
k
E_p
E_s
Sample Rotation
$\theta < \theta_c$
i
Sample Orientation:
45° 135° 225° 315° Average
LDS
Wavelength (nm)

Figure S2: In contrast to the PWs (Main Text, Figure 1), the LDS of a single nanorod varies as a function of sample orientation. (a) Schematic of a common experiment design for determining the orientation of single nanorods. A dark-field condenser illuminates the nanorods with unpolarized light and the light scattered from the rod is passed through a rotating linear polarizer before reaching a detector. (b) Scattering spectra of a single nanorod as the linear polarizer is rotated through 180° in 30° steps. The nanorod's scattering is greatest when the orientation of the long axis of the rod is well-aligned with the angle δ of the linear polarizer; in this case, at 30° and 60° (yellow and orange lines). When the polarizer's angle is perpendicular to the rod's orientation, the scattering is strongly suppressed; here at 120° and 150° (green and teal lines). The inset shows a representative scanning electron microscope (SEM) image of a lithographically fabricated nanorod oriented at a ~45° angle. (c) Same as (b) but for a nanorod aligned perpendicular with respect to that shown in (b), at ~135°. (d) Integrated scattering at the longitudinal surface plasmon resonance of the nanorod in (b) as a function of the linear polarizer angle. The scattering follows a $\cos^2(\delta - \zeta)$ dependence where ζ is the orientation angle of the long axis of the nanorod in the sample plane.[1] Fitting the data to a $\cos^2(\delta - \zeta)$ function allows the assignment of the nanorod's orientation at 45°. (e) Fitting the integrated scattering of the nanorod in (c) to a $\cos^2(\delta - \zeta)$ dependence as well confirms that this nanorod is oriented perpendicular to the nanorod in (b) at 133°. (f) LDS spectrum of the same nanorod in (b) calculated as $LDS(\lambda) = Scatt(120°) - Scatt\,(30°)$. We chose this definition to be consistent with the Main Text in which a nanorod oriented at 45° would have negative LDS if calculated as $LDS(\lambda) = Scatt(E_s = -E_p) - Scatt(E_s = E_p) = Scatt(135°) - Scatt(45°)$. Here, as we only measured LDS in 30° increments, we subtract the spectrum acquired at the angle of maximum scattering from that acquired at the angle of minimum scattering, yielding a negative LDS spectrum. (g) In contrast,

the LDS spectrum of the nanorod oriented at 133° is positive at all wavelengths. In summary, two nanorods oriented orthogonally with respect to each other yield LDS spectra of opposite signs. (h) Alternative LDS experiment design in which the incident light is linearly polarized, here at $E_s = \pm E_p$, and the sample is rotated, rather than the linear polarizer. (i) LDS spectra of two single nanorods measured at each sample orientation. The LDS inverts each time the sample is rotated 90°. For example, the LDS of the nanorod shown in the left panel of (i) is negative at 45° (blue curve) and inverts to a positive spectrum when the sample is rotated 90° to an orientation of 135° (orange curve). In total, the LDS of anisotropic particles strongly depends on sample orientation and generally averages to zero in the orientation-averaged LDS (black curves) if the LDS is measured at four orthogonal sample orientations.

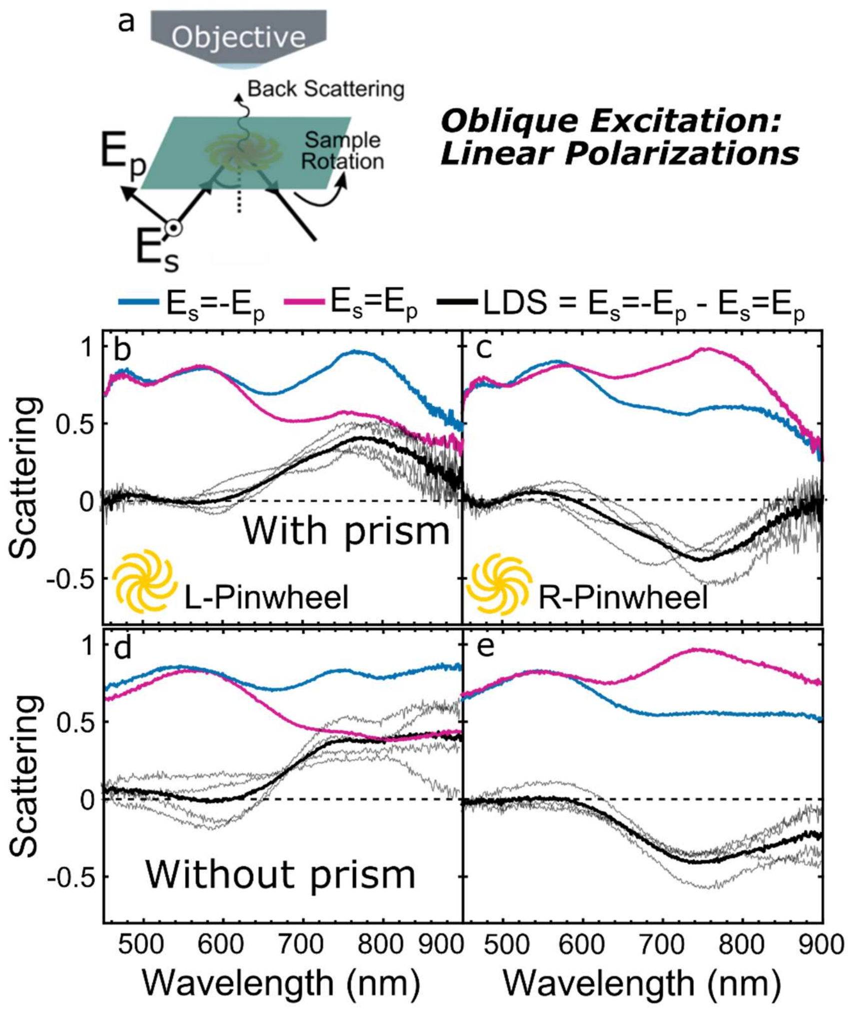

Figure S3: The orientation-independent LDS is not due to total internal reflection (TIR) and associated trochoidal polarizations that may be present when we use light refracted at a high angle.[3] (a) Experimental geometry for oblique-incident linearly polarized excitation without the use of a prism. In this geometry, $E_s = \pm E_p$ light directly illuminates the PW without passing through any interfaces. In contrast, our typical scattering geometry presented in Main-Text Figure 1 offers a higher signal-to-noise ratio but involves coupling the incident light into a prism and refracting it at a high angle through the glass-air interface. (b) and (c) Solid lines: scattering from a single L- and R-PW under oblique incident, prism coupled, $E_s = -E_p$ and $E_s = E_p$ light, averaged over the four orthogonal sample orientations. Solid black line: mean LDS spectrum. The

light gray lines depict the LDS spectra measured at each orientation. (d) and (e) same as (b) and (c) but for oblique-incident light that directly irradiates the PWs without refracting through any interfaces. The general reproducibility of our results acquired without a prism demonstrates that orientation-independent LDS is not due to any polarization distortion occurring from refracting the incident light through a glass-air interface.

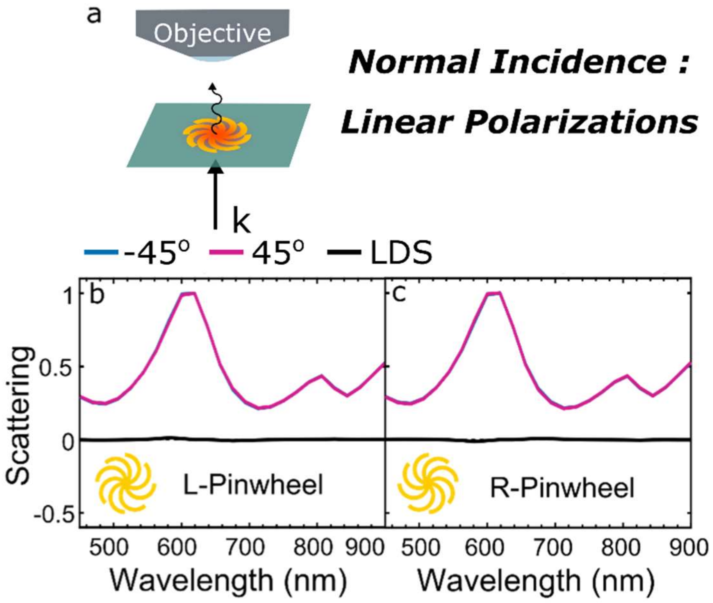

Figure S4: Simulations of the LDS of PWs when irradiated with ±45° linearly polarized light directed at a normal incidence confirm that the PWs behave isotropically under illumination that maintains the rotational symmetry of the system. (a) Schematic of the normal-incidence simulation geometry. (b) Scattering spectra of an L-PW under ±45° linearly polarized light. (c) Same as (b) but for an R-PW.

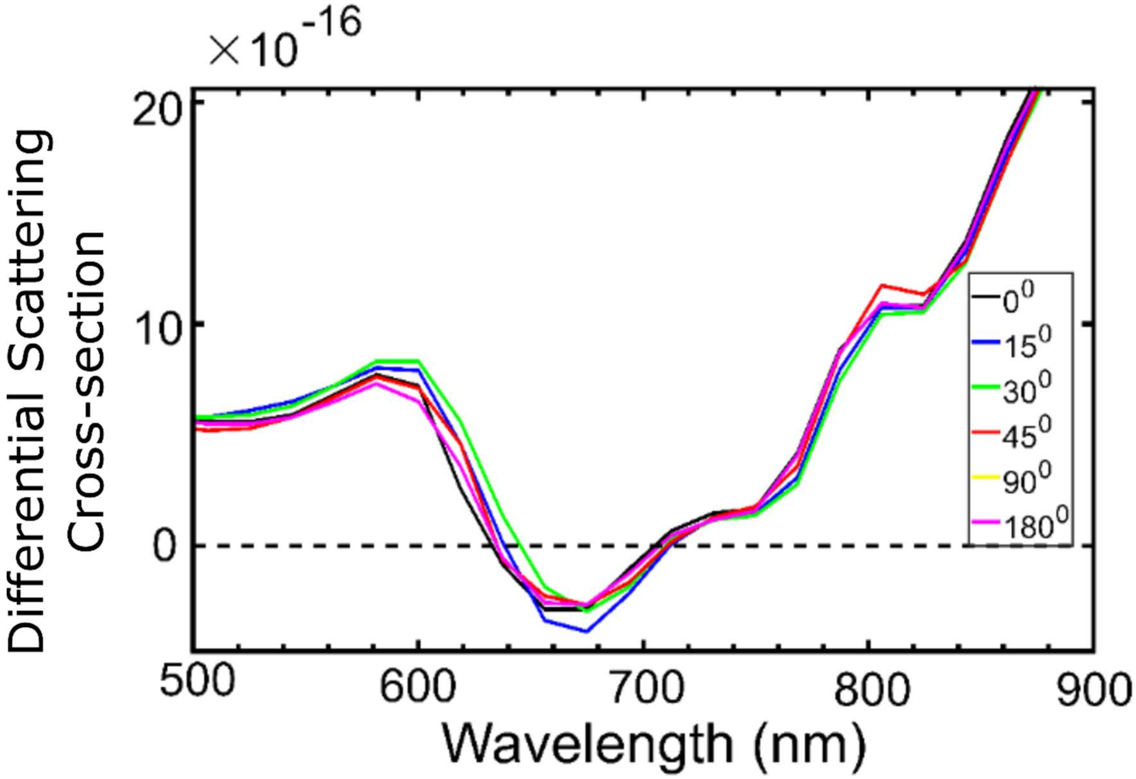

Figure S5: Simulations of the differential scattering cross-sections of an L-PW at 0°, 15°, 30°, 45°, 90°, and 180° sample orientations confirm that the LDS is independent of the PW's orientation with respect to *k*, consistent with experimental results presented in Main-Text Figure 1.

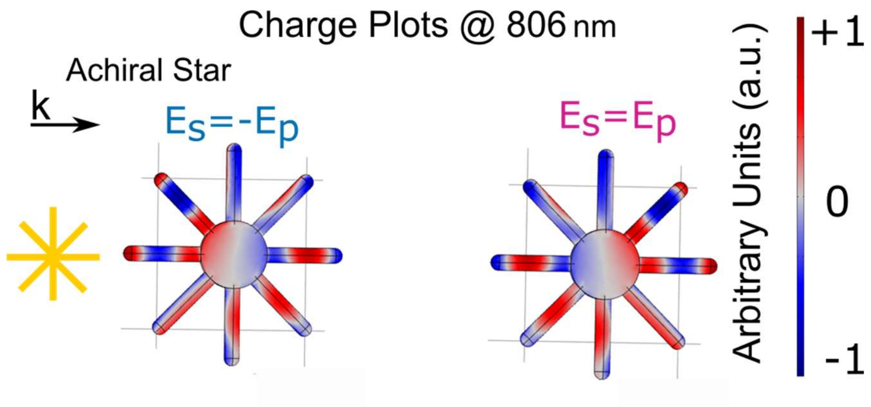

Figure S6: Simulated charge plots of the achiral star confirm a symmetric charge distribution under both incident polarizations. Simulated light-induced charge density of the achiral star at 806 nm under $E_s = \pm E_p$ excitation. Unlike the PWs (Main Text, Figure 5), the stars do not exhibit preferential charge accumulation on two arms under only one polarization. Instead, the charge plots are exactly mirror-symmetric under $E_s = \pm E_p$ light, leading to no preferential interaction for either linear polarization.

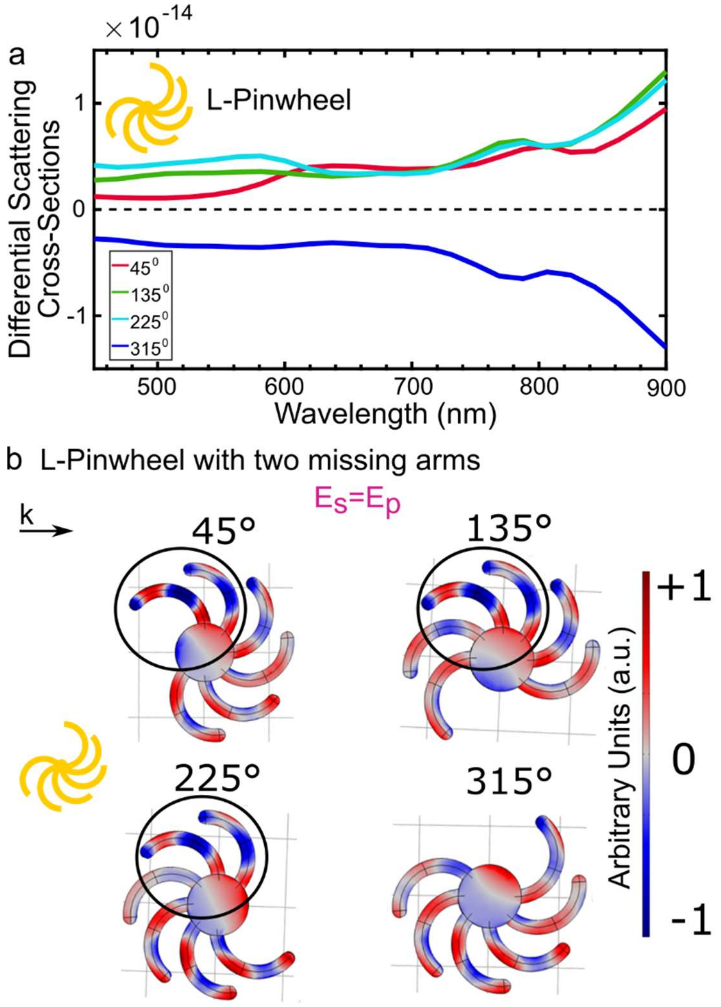

Figure S7: Simulations of the LDS for the L-PW shown in Main-Text Figure 6c with two missing arms demonstrate that the LDS sign inverts at the 315° sample orientation, in agreement with our experimental observations, with simulated charge plots of the 6-arm L-PW elucidating the origin of the inverted sign. (a) Linear differential scattering cross-sections simulated for $E_s = \pm E_p$ incident light for sample orientations of 45°, 135°, 225°, and 315°. In contrast to the negative differential scattering at 315°, at the 45°, 135°, and 225° polar angles, the LDS is positive in sign and consistent with that of an 8-arm L-PW (Main Text, Figure 1). (b) Simulated charge plots for the L-PW with two missing arms under $E_s = E_p$ polarization at 806 nm for each of the four polar angles describing the sample orientation. The PWs with missing arms

exhibit charge accumulation on the top two circled arms at orientations of 45°, 135°, and 225°. At 315°, the top two arms are missing, yielding an even charge distribution over the entire PW and hence effectively altering the scattering efficiency. This difference causes the inversion of the LDS for the L-PW, shown in part a, and in Main-Text Figure 6.

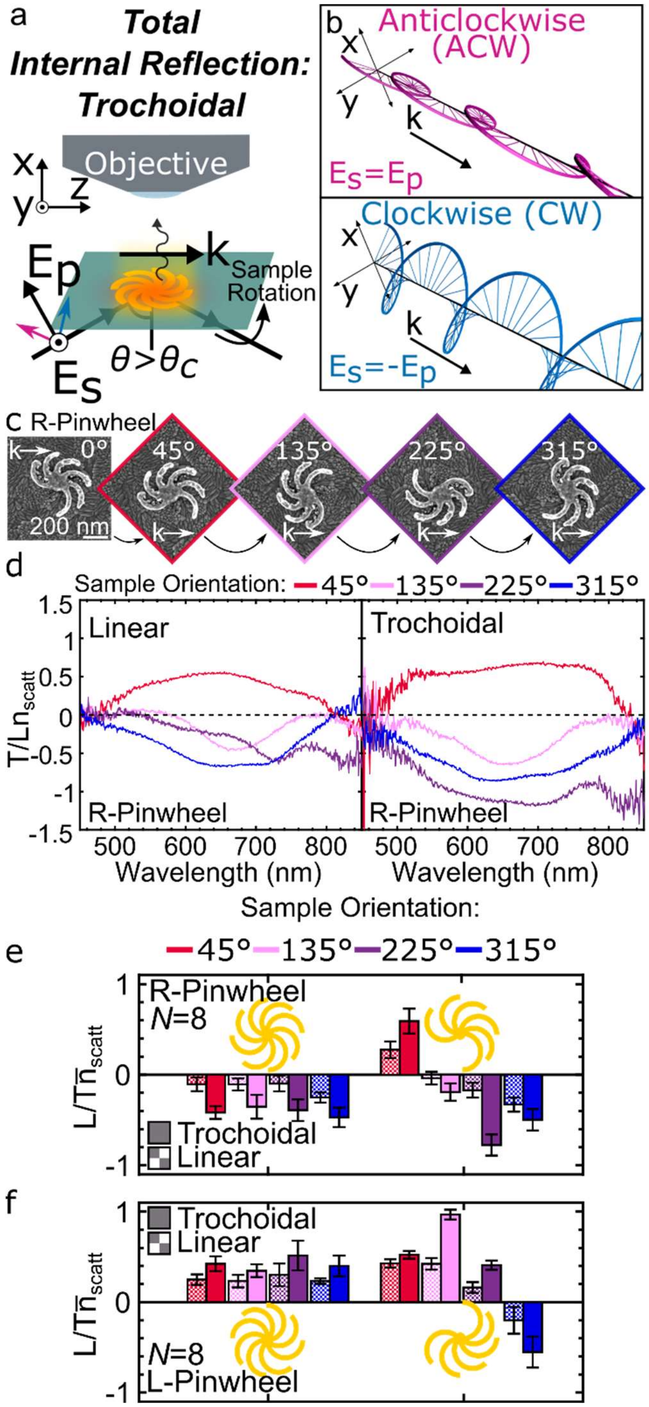

Figure S8: At all orientations, PWs with two missing arms exhibit larger trochoidal differential scattering (TDS) than LDS. (a) TIR experiment geometry with linearly polarized light incident at an azimuthal angle (θ) that exceeds the critical angle (θ_c). In both TDS and LDS

measurements, the incident light is linearly polarized at $E_s = \pm E_p$ ($\pm 45°$), shown by the pink and blue arrows, respectively. The main difference between the two measurements is that in TDS experiments, the incident light undergoes TIR, which produces an evanescent wave with cycloid-like trochoidal polarizations rotating in clockwise (CW) and anticlockwise (ACW) directions.[3] $\boldsymbol{k}$ is the real component of the evanescent wave vector and the curved arrow at the edge of the glass slide depicts the direction in which the sample is rotated to characterize TDS at multiple orientations relative to $\boldsymbol{k}$. (b) Instantaneous electric field distributions of the evanescent wave produced by TIR of $E_s = \pm E_p$ light. With $E_s = E_p$ ($E_s = -E_p$) incident linear polarization, the electric field rotates in an ACW (CW) direction at an approximately $45°$ ($-45°$) angle tilted out of the sample (y, z) plane. TDS, then, is the differential scattering under CW and ACW polarizations.[4] (c) SEM image of an R-PW rotated through each of the sample orientations. (d) Ln_{scatt} (left) and Tn_{scatt} (right) calculated at each orientation for the same R-PW with two missing arms. Note that Tn_{scatt} is calculated analogously to Ln_{scatt} as $Tn_{scatt}(\lambda) = \frac{Scatt(CW) - Scatt(ACW)}{\frac{1}{2}(Scatt(CW) + Scatt(ACW))}$. (e) Grouped bar chart for the spectrally integrated, mean $L\bar{n}_{scatt}$ and $T\bar{n}_{scatt}$ (see Main Text equation 2 for definition), calculated at each sample orientation for R-PWs with no missing arms and 2 missing arms, corresponding to the cartoon geometry above the group. Error bars correspond to the standard deviation of the mean $L/T\bar{n}_{scatt}$ for each geometry and orientation. Bars corresponding to mean $T\bar{n}_{scatt}$ are solid in color, while those corresponding to $L\bar{n}_{scatt}$ are checkered. N: number of PWs considered in each bar. (f) Same as (e) but for the L-PWs. Considering the mean $L\bar{n}_{scatt}$ and $T\bar{n}_{scatt}$ from 8 PWs characterized for each geometry demonstrate that at all orientations, the measured $T\bar{n}_{scatt}$ exceeds the $L\bar{n}_{scatt}$.

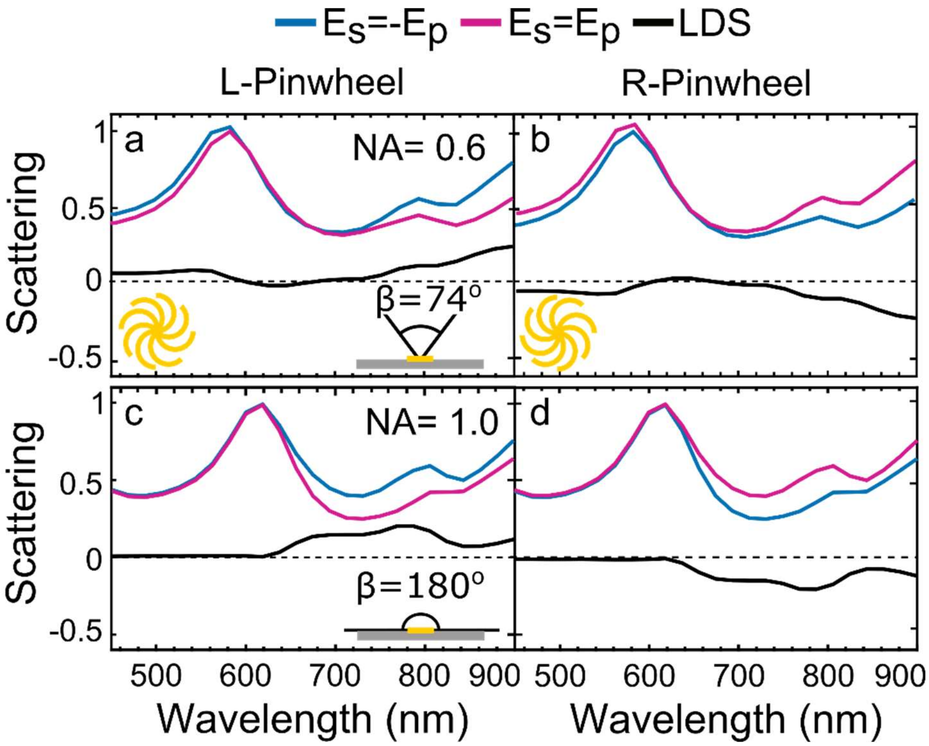

Figure S9: The magnitude of the measured LDS of the PWs is not significantly impacted by the numerical aperture (NA) of the objective used to collect the scattered light. (a) and (b) Simulated scattering spectra of the L-PW and R-PW, respectively, under oblique-incident $E_s = \pm E_p$ linear polarizations collected with an angular aperture (β) of 74° (NA of 0.6), matching the objective used in experiment. (c) and (d) same as (a) and (b) but simulated for light collected with an angular aperture of 180° (NA= 1.0). Although the spectral shape of the scattering and the LDS depends on the NA of the objective, the magnitude and sign of the LDS is sufficiently captured with a lower NA objective.

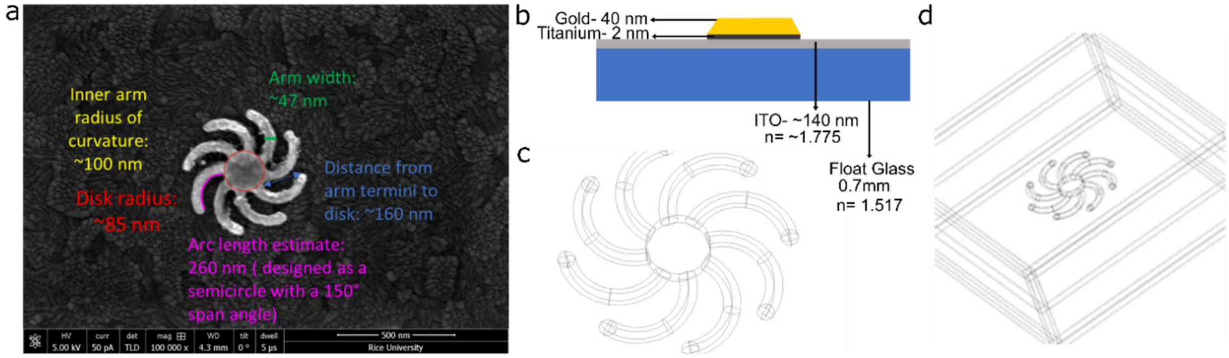

Figure S10: (a) Enlarged SEM image of an L- PW showing the geometric parameters based on which the simulated structures were modeled. (b) Schematic representation of the background medium layers on which the structure rests. (c) PW geometry modeled in COMSOL Multiphysics and (d) simulation region with the background medium layers and boundaries.

REFERENCES

1. Sönnichsen, C, Alivisatos, AP Gold nanorods as novel nonbleaching plasmon-based orientation sensors for polarized single-particle microscopy. Nano Lett. **2005**, 5, 301-304.
2. Marchuk, K, Ha, JW, Fang, N Three-Dimensional High-Resolution Rotational Tracking with Superlocalization Reveals Conformations of Surface-Bound Anisotropic Nanoparticles. Nano Lett. **2013**, 13, 1245-1250
3. Bliokh, K. Y.; Bekshaev, A. Y.; Nori, F., Extraordinary Momentum and Spin in Evanescent Waves. Nat. Commun. **2014**, 5, 3300.
4. McCarthy, L. A.; Smith, K. W.; Lan, X.; Hosseini Jebeli, S. A.; Bursi, L.; Alabastri, A.; Chang, W.-S.; Nordlander, P.; Link, S., Polarized Evanescent Waves Reveal Trochoidal Dichroism. Proc. Natl. Acad. Sci. U. S. A. **2020**, 117, 16143-16148.
